# Attentional capture by emotional faces in adolescence

**DOI:** 10.2478/v10053-008-0134-9

**Published:** 2013-06-17

**Authors:** Jillian Grose-Fifer, Andrea Rodrigues, Steven Hoover, Tina Zottoli

**Affiliations:** Department of Psychology, John Jay College of Criminal Justice, The City University of New York, USA; The Graduate Center, Forensic Psychology Doctoral subprogram, The City University of New York, USA

**Keywords:** adolescence, cognitive control, flanker task, affective, non-affective, risk taking

## Abstract

Poor decision making during adolescence occurs most frequently when situations are emotionally charged. However, relatively few studies have measured the development of cognitive control in response to emotional stimuli in this population. This study used both affective (emotional faces) and non-affective (letter) stimuli in two different flanker tasks to assess the ability to ignore task-irrelevant but distracting information, in 25 adults and 25 adolescents. On the non-emotional (letter) flanker task, the presence of incongruent flanking letters increased the number of errors, and also slowed participants’ ability to identify a central letter. Adolescents committed more errors than adults, but there were no age-related differences for the reaction time interference effect in the letter condition. Post-hoc testing revealed that age-related differences on the task were driven by the younger adolescents (11-14 years); adults and older adolescents (15-17 years) were equally accurate in the letter condition. In contrast, on the emotional face flanker task, not only were adolescents less accurate than adults but they were also more distracted by task-irrelevant fearful faces as evidenced by greater reaction time interference effects. Our findings suggest that the ability to self-regulate in adolescents, as evidenced by the ability to suppress irrelevant information on a flanker task, is more difficult when stimuli are affective in nature. The ability to ignore irrelevant flankers appears to mature earlier for non-affective stimuli than for affective stimuli.

## Introduction

As adolescents transition from childhood to adulthood, there is considerable neural and cognitive development that helps to foster their growing independence. Many aspects of executive function show linear improvements with age, which suggests that as adolescents grow older they should be increasingly capable of controlling their thoughts and actions (for a review, see Crone, 2009). However, effective decision making in real-life situations depends on the complex interaction among cognitive, emotional, and psychosocial processes, many of which are still developing during adolescence (for reviews, see Blakemore & Choudhury, 2006; Gardner & Steinberg, 2005; Luna, 2009; Rivers, Reyna, & Mills, 2008; Steinberg, 2007, 2008). Adolescent decision making is frequently compromised by heightened sensitivities to emotion, peer presence, novelty and reward, and by increased sensation seeking (for reviews, see Casey, Jones, & Hare, 2008; Casey et al., 2010; Crone, 2009; Somerville, Jones, & Casey, 2010; Steinberg, 2008, 2010). This underscores the fact that self-regulation is a complex phenomenon that requires many different aspects of cognitive control. Several neurobiological studies have posited that adolescents find it more difficult to override the increased push from affective processes because the cortical control systems, predominantly in the prefrontal cortex (PFC), have a more prolonged developmental trajectory than those involved in processing incentives and emotions (Casey et al., 2008, 2010; Chein, Albert, O’Brien, Uckert, & Steinberg, 2011; Padmanabhan, Geier, Ordaz, Teslovich, & Luna, 2011; Van Leijenhorst et al., 2010).

A considerable number of studies have focused on assessing cognitive control in normally developing adolescents using a variety of abstract laboratory tasks involving conflict between stimuli (for a review, see Luna, 2009). A common feature of these paradigms is that the participant has to complete a task while ignoring or inhibiting a response to highly distracting, but goal-irrelevant information. These include go/no-go paradigms (Casey et al., 1997; Galvan et al., 2005; Hooper, Luciana, Conklin, & Yarger, 2004; Lamm, Zelazo, & Lewis, 2006), the word-color Stroop test (Adleman et al., 2002), anti-saccade tasks (Luna et al., 2001), and flanker tasks (Davies, Segalowitz, & Gavin, 2004; Ladouceur, Dahl, & Carter, 2004, 2007; Santesso & Segalowitz, 2008). Under optimal conditions, performance on these decontextualized,abstract tasks, often referred to as “cool” cognitive tasks (Lazarus & Smith, 1988), frequently has been shown to be adult-like by 15 to 16 years of age (e.g., Ladouceur et al., 2007; Luna, Padmanabhan, & O’Hearn, 2010; Santesso & Segalowitz, 2008). In contrast, studies that have assessed how cognitive control in adolescence is influenced by affective factors, such as incentive processing (Burnett, Bault, Coricelli, & Blakemore, 2010; Cauffman et al., 2010; Crone, Bullens, van der Plas, Kijkuit, & Zelazo, 2008; Geier & Luna, 2009; Padmanabhan et al., 2011; Van Leijenhorst et al., 2010), the presence of peers (Chein et al., 2011; Gardner & Steinberg, 2005), or the use of emotional faces (Hare et al., 2008; Monk et al., 2003; Somerville, Hare, & Casey, 2011), have shown that relative to adults, adolescent performance is disproportionately degraded on tasks where these affective or “hot” cognitive processes (Lazarus & Smith, 1988) are engaged.

Problematically, relatively few studies have compared hot and cool cognitive control in the same sample of adolescents. Of the studies that have, the majority (e.g., Hooper et al., 2004; Lamm et al., 2006; Overman et al., 2004; Prencipe et al., 2011; Steinberg, 2010; van Duijvenvoorde, Jansen, Visser, & Huizenga, 2010) have shown that performance on executive function tasks has a more prolonged developmental trajectory when a task has an affective or motivational component to it (e.g., Iowa Gambling Task [IGT] or a modification thereof) than when it is non-affective or more abstract in nature (e.g., color-word Stroop or Wisconsin Card Sort Task). However, hot and cool executive function task comparisons may be confounded by the fact that the tasks themselves frequently have very different requirements. For example, the Stroop task requires participants to inhibit the natural tendency to read words instead of responding to another feature of the stimulus (such as color), whereas the IGT requires that participants use feedback to figure out which card decks are more advantageous in the long run.

Chein et al. (2011) avoided the task-related problems associated with these executive function studies by using peer presence to increase the affective element of a virtual driving task. When driving alone, both teenagers and adults performed comparably; however, in the hotter version of the task (i.e., when peers were present), adolescents took more risks and were less likely to stop at red lights than adults. Although this within-subjects study provides important empirical support for the anecdote that adolescents are more likely to take risks in the presence of peers than when alone, the difficulties associated with recruiting a peer may limit the utility of this design in future investigations.

The objective of the current study was to help address some of the issues outlined above, by comparing cognitive control in the same sample of adolescents and adults using a hot and a cool version of the flanker task (Eriksen & Eriksen, 1974). The traditional cool version of the flanker task requires that the participant identify a central target (typically a letter or an arrow) that is flanked either by similar stimuli (congruent condition) or dissimilar stimuli (incongruent condition). Participants are generally faster and more accurate in identifying the target in congruent trials, whereas incongruent flankers distract attention away from the task at hand, which results in an increased reaction time for target identification (Eriksen & Eriksen, 1974).

In our study we used emotional faces to manipulate the motivational salience of the task, which is a similar approach to that of Casey and colleagues who used face stimuli in go/no-go tasks to measure cognitive control in adolescents (Hare et al., 2008; Somerville, Hare, & Casey, 2011). They found that adolescents (*M* = 15.9 years) made more commission errors than adults (*M* = 23.7 years) or children (*M* = 9.5 years) on no-go trials for happy faces relative to calm faces (Somerville, Hare, & Casey, 2011). In other words, there was an adolescent-specific decrement in the ability to inhibit responses to appetitive stimuli. On the other hand, they also reported that both adolescents (*M* = 16.0 years) and children (*M* = 9.1 years) made slower go-responses to fearful faces compared to adults (*M* = 23.9 years; Hare et al., 2008), suggesting that cognitive control of approach behaviors to aversive stimuli follows a more linear developmental time course. Therefore, development appears to differentially moderate the effect of appetitive and aversive face stimuli on behavioral measures of cognitive control of approach and avoidance behaviors. However, not only can cognitive control be measured in a variety of ways but performance on different measures may have temporally distinct developmental trajectories (Bunge, Hazeltine, Scanlon, Rosen, & Gabrieli, 2002). This underscores the need to use paradigms other than go/no-go tasks to better understand how cognitive control is affected by emotional faces in adolescents. Therefore, the current study examined how emotional faces affect the ability to ignore extraneous information from competing choices in a flanker task, rather than in a go/no-go task. Performance on a flanker task differs from that in a go/no-go task, in that it relies on selective (forced choice) rather than nonselective inhibition (van Boxtel, van der Molen, Jennings, & Brunia, 2001), and the ability to ignore goal-irrelevant stimulus interference, rather than the suppression of a prepotent response (Nigg, 2000). Therefore, our study provides additional information about the interaction between affective cues and cognitive control in adolescents.

Behavioral performance on traditional flanker tasks has been shown to be adult-like by mid-adolescence (Davies et al., 2004; Ladouceur et al., 2004, 2007; Santesso & Segalowitz, 2008), however, the neural structures that mediate this response are still developing (Davies et al., 2004; Ladouceur et al., 2004, 2007; Rubia, Smith, Taylor, & Brammer, 2007; Santesso & Segalowitz, 2008; Velanova, Wheeler, & Luna, 2008). Therefore, inefficiencies in response monitoring in adolescents might only become apparent if the task-demands are increased in some way (Santesso & Segalowitz, 2008). Presumably this could be achieved by using a secondary task to increase working memory load (cf. Lavie & De Fockert, 2005) or by increasing the motivational significance of the stimuli. However, to our knowledge, neither approach has been used in a flanker task with adolescents.

Given the extant literature that shows that behavioral performance on purely cognitive tasks matures more quickly than that on affective tasks (Hooper et al., 2004; Lamm et al., 2006; Overman et al., 2004; Prencipe et al., 2011; Steinberg, 2010; van Duijvenvoorde et al., 2010),we hypothesized that we would replicate the findings of others (Davies et al., 2004; Ladouceur et al., 2004, 2007; Santesso & Segalowitz, 2008) that adolescents (*M* = 15 years) would be able to ignore non-emotional distracting stimuli on a flanker task as effectively as adults. However, we also predicted that teenagers would do more poorly than adults when faced with the additional challenge of ignoring emotional faces on a flanker task. Ochsner and colleagues have shown that for adults, overriding conflict experienced in affective and nonaffective versions of a word flanker task activated several common areas, including the dorsal anterior cingulate cortex (ACC), dorsolateral PFC, and posterior medial frontal cortex. In contrast, conflict in the affective condition selectively activated the rostral medial PFC (Ochsner, Hughes, Robertson, Cooper, & Gabrieli, 2009). Therefore, our hypothesis that adolescents would find emotional faces more distracting than would adults, rests on the following assumption: Emotional stimuli should activate cortical and subcortical areas associated with affective processing, thereby increasing the amount of cognitive control needed to ignore the irrelevant flankers. Since the ACC and other areas of the PFC that are important for cognitive control on flanker tasks (Ochsner et al., 2009) are still maturing during adolescence (including the ventromedial PFC, which is implicated in emotional control; cf. Davies et al., 2004; Giedd, 2004; Ladouceur et al., 2004, 2007; Rubia et al., 2007; Santesso & Segalowitz, 2008; Velanova et al., 2008), this additional demand should lead to poorer performance for adolescents relative to adults.

To allow comparisons with other studies of cognitive control in adolescents, our design used both letters (non-affective condition) and emotional faces (affective condition) as stimuli in two separate flanker tasks (for a similar design in adults, see Munro et al., 2007). As far as we are aware, only a few other studies have used emotional faces in flanker tasks, and these have all used adult samples (Fenske & Eastwood, 2003; Moser, Huppert, Duval, & Simons, 2008; Munro et al., 2007). Given that face perception is still developing during adolescence (for reviews, see Batty & Taylor, 2006; Blakemore, 2008; Herba & Phillips, 2004; Scherf, Behrmann, & Dahl, 2011; Somerville, Fani, & McClure-Tone, 2011), we chose to limit our stimuli to facial expressions that should be relatively easy for adolescents to differentiate (i.e., happy and fearful). Specifically, we did not use neutral faces because others have shown that these may be more difficult for adolescents and children to identify (Herba & Phillips, 2004; K. M. Thomas et al., 2001; L. A. Thomas, De Bellis, Graham, & LaBar, 2007). Nor did we use calm faces because when we started data collection these had not been widely used in children and adolescents (though see Hare et al., 2008; Somerville, Hare, & Casey, 2011).

Fenske and Eastwood (2003) demonstrated that in adults, negative faces captured attention more effectively than positive faces in a schematic face flanker task. However, both fearful faces (Monk et al., 2003) and happy faces (Joormann, Talbot, & Gotlib, 2007; Somerville, Hare, & Casey, 2011) have been shown to be more distracting to normally developing adolescents than to adults. In light of this, we did not hypothesize as to how the valence of the faces would affect adolescent performance, merely that adolescents would be disproportionately more distracted by incongruent flankers in the face task than would adults. In addition, since it is possible that gender differences in face processing abilities (for reviews, see McClure, 2000; Scherf et al., 2011; Somerville, Fani, & McClure-Tone, 2011) could affect distractibility, the influence of gender was also examined in relation to performance on the flanker tasks.

## Method

### Participants

Twenty-five adults (12 males) between 23 and 35 years of age (*M* = 28.08years, *SD* = 3.24) and 25 adolescents (14 males) between 11 and 17 years of age (*M* =15.00 years, *SD* = 1.66) took part in this study. The participants were recruited via advertisement on Craigslist.com, and by flyers posted in local high schools, at John Jay College, and in the surrounding community. All participants had normal or corrected-to-normal vision, and no history of neurological or psychological disorders. Informed consent was obtained prior to the start of the study. For adolescent participants, both informed parental consent and child assent were obtained. All participants received $15 for their time.

### Procedure

In order to compare the ability to ignore distracters across non-emotional and emotional conditions, participants performed both a traditional letter flanker task (Eriksen & Eriksen, 1974) and an emotional face flanker task. Each flanker task began with a practice session, which was repeated until the participant felt ready to begin the task. Stimuli were presented on a computer screen (Dell 1908 Flat Panel LCD monitor) using E-prime 2.0 software (Psychology Software Tools Inc.) and the participant sat at a distance of 65 cm from the screen.

### Materials

#### Letter Flanker Stimuli

On each trial in the letter flanker task, a central letter was flanked on either side by two letters. Stimuli were presented in one of two conditions: congruent or incongruent. In the congruent condition, all the letters were identical: *HHHHH* or *SSSSS*. In the incongruent condition, the flanking letters did not match the central letter: *HHSHH* or *SSHSS*. Participants had to identify the central letter by pressing the relevant mouse button (e.g., left button for H and right for S). Participants were encouraged to be as fast and as accurate as possible. The target/mouse assignation was counterbalanced across participants. Each stimulus was displayed for 200 ms and was preceded by a fixation cross that was present for 500 ms. The inter-trial interval (ITI) was 1,250 ms. The average angular subtense of the entire stimulus (e.g., HHSHH) was 2.3 (vertical) × 8 deg (horizontal). A total of 144 trials were shown over four blocks, half of these were incongruent. Participants were encouraged to rest between blocks.

#### Emotional Face Stimuli

**Figure 1.**
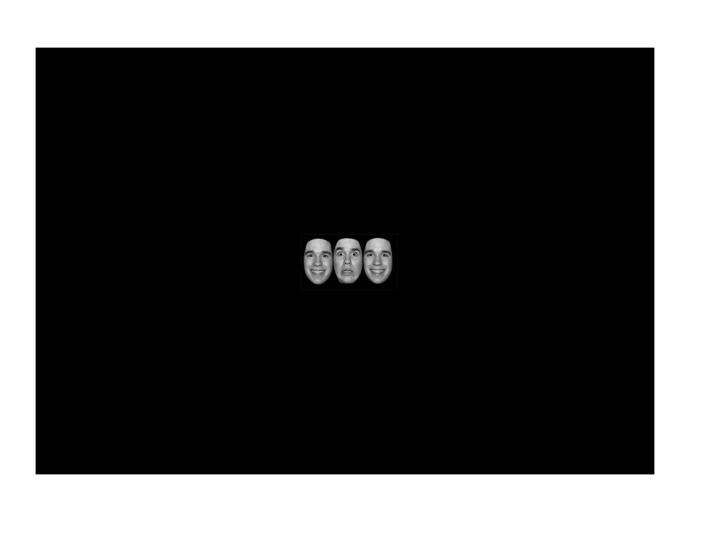
Example of a fearful incongruent stimulus.

Photos of 18 different people (nine female, nine male) showing happy or fearful emotional expressions were chosen from the NIMSTIM face stimuli set (Tottenham et al., 2009). This relatively large number of faces was used to try and to prevent habituation effects from occurring too rapidly. Faces were shown in one of four conditions: (a) happy congruent (happy face flanked by happy faces), (b) happy incongruent (happy face flanked by fearful faces), (c) fearful congruent (fearful face flanked by fearful faces), and (d) fearful incongruent (fearful face flanked by happy faces). The faces were small and tightly arranged in order to produce maximum interference; the angular subtense of the three faces together was the same as that for the letter stimuli. Participants had to identify the central face by pressing the relevant mouse button (e.g., left button for happy and right for fearful faces). The target/mouse assignation was counterbalanced across participants. Participants were encouraged to be as fast and as accurate as possible. Figure 1 shows an example of a fearful incongruent trial. The same face was always used within a trial (i.e., the target and flankers were always of the same person). This was done to eliminate possible confounds concerning attentional capture due to low-level visual processing differences between the three faces. Each stimulus was presented for 400 ms and was preceded by a fixation cross, which was present for 500 ms. The ITI was 2.8 s. The duration and ITI were made considerably longer than the letter trials, because our pilot study showed that many adult participants were unable to perform the task at shorter durations and ITIs. There were 36 trials in each condition. The trials were pseudo-randomized and presented in 12 blocks, such that the same face was never repeated within a block; all conditions were present three times within a block. Each trial appeared only once during the first half of the experiment, and was then repeated in the second set of blocks. Participants were encouraged to rest between blocks.

### Analysis

The mean reaction time (RT) and the percentage of correct responses (accuracy) were calculated for each condition for each participant. For correct trials on each condition, RT outliers (greater or less than three standard deviations from mean) were removed (i.e., 3.5% of trials for adolescents and 3.6% of trials for adults). Both RT for correct trials, and accuracy were then entered into separate repeated measures ANOVAs, both for the letter task using a within-subjects factor of Congruency (congruent, incongruent) and between-subjects factors of Age Group (adolescent, adult) and Gender (male, female); and for the emotional face task with within-subjects factors of Target (happy, fearful), Congruency (congruent, incongruent), and between-subjects factors of Age Group (teen, adult) and Gender (male, female). As is common in flanker task analysis (Eriksen & Eriksen, 1974), cognitive control efficiency was measured by the amount of interference that the incongruent flankers produced by subtracting the congruent RT from the incongruent RT for each target. Consequently, an interference effect score of zero indicated that for a given target, the incongruent flankers did not slow the participant in identifying the central target. ANOVAs with the interference effect as the dependent variable were used to clarify the nature of any congruency, target, and/or age inter-actions on RT.

## Results

### Accuracy

**Figure 2.**
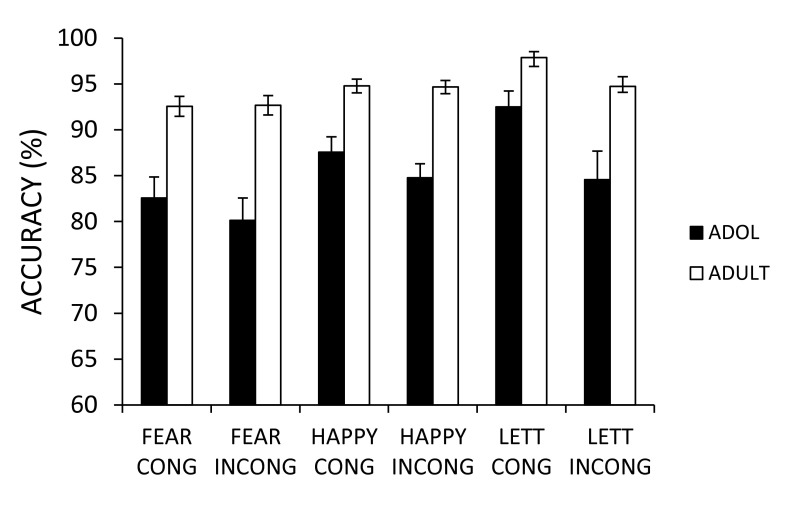
Mean accuracy (% correct) for each stimulus category. ADOL = adolescent. CONG = congruent. INCONG = incongruent. LETT = letter.

Figure 2 shows the mean accuracy (M) and the standard error (SE) for each condition for both adults and adolescents in each of the tasks.

#### Non-affective letter task

Accuracy was poorer on incongruent (*M* = 89.6%, *SD* = 12.7) com-pared to congruent (*M* = 95.2%, *SD* = 7.0) trials, *F*(1, 46) = 24.61, *p* < .001, η_p_^2^ = .36. Adolescents were less accurate (*M* = 88.5%, *SD* = 13.1) than adults (*M* = 96.3%, *SD* = 4.6), *F*(1, 46) = 9.42, *p* = .004, η_p_^2^ = .17. There was no effect of gender (p > .1, η_p_^2^ < .001) and no interactions with Gender (for all tests, p > .1, η_p_^2^ < .02). The Age Group by Congruency interaction was marginally significant, *F*(1, 46) = 3.97, *p* = .05, η_p_^2^ = .08; the incongruent condition increased the number of errors proportionately more for adolescents than for adults.

##### Post-hoc analyses

We were somewhat surprised that adolescents were not as accurate as the adults on the letter task, given that others have shown that performance on non-affective flanker tasks reaches adult-like maturity by 15 years of age (Davies et al., 2004; Ladouceur et al., 2004, 2007; Santesso & Segalowitz, 2008). To investigate this further, we collapsed across gender and subdivided the data into three age groups: younger adolescents (11-14 years, *n* = 9), older adolescents (15-17 years, *n* =16), and adults (25-35 years, *n* = 25) and found that accuracy on congruent trials had a nonlinear relationship with age, linear term, *F*(1, 47) = 23.2, *p* < .001, quadratic term, *F*(1, 47) = 4.43, *p* = .041. A nonlinear relationship with age was also found for accuracy on the incongruent trials, linear term, *F*(1, 47) = 32.5, *p* < .001, quadratic term, *F*(1, 49) = 8.4, *p* = .006. These nonlinear relationships were clarified by post hoc tests of pairwise comparisons, which showed that younger adolescents were less accurate (*M* = 79.0%, *SD* = 13.5) than both older adolescents (*M* = 93.9%, *SD* = 5.4, *M*_diff_ = 14.9%, *p* = .03) and adults (*M* = 96.3%, *SD* = 4.0, *M*_diff_ = 17.3%, *p* = .01). In contrast, there was no significant difference in accuracy between older adolescents and adults (*M*_diff_ = 2.4%, *p* = .33). Thus, the age-related effects on accuracy on the letter task were driven by the younger adolescents. These data support the hypothesis that the ability to accurately ignore extraneous non-affective information matures during adolescence, and appears adult-like by 15-17 years.

#### Affective face task

Adolescents were less accurate (*M* = 83.8%, *SD* = 10.4) than adults (*M* = 93.7%, *SD* = 4.6) on the face task, *F*(1, 46) = 30.79, *p* < .001, η_p_^2^ = .40. Accuracy was lower for fearful (*M* = 87.0%, *SD* = 10.7) than for happy targets (*M* = 90.4%, *SD* = 7.6), *F*(1, 46) = 11.72, *p* = .0001, η_p_^2^ = .20, and lower for the incongruent (*M* = 88.1%, *SD* = 9.8) compared to the congruent (*M* = 89.4%, *SD* = 9.1) trials, although this effect was only marginally significant, *F*(1, 46) = 3.72, *p* = .06, η_p_^2^ = .07. There was no effect of gender (p > .1, η_p_^2^ < .001) and no interactions with Gender (for all effects, *ps* > .1, η_p_^2^ < .02).

##### Post-hoc analyses

Again, we examined these data using the three age group approach described above, and found that accuracy improved linearly with age for each of the four face target conditions (all linear terms *p* < .001, all quadratic terms p > .2). Therefore, in contrast to the results for the non-affective task, the ability to respond accurately to emotional face targets appears to improve linearly with age across adolescence, and is not yet adult-like by 15-17 years.

### Reaction time

**Figure 3.**
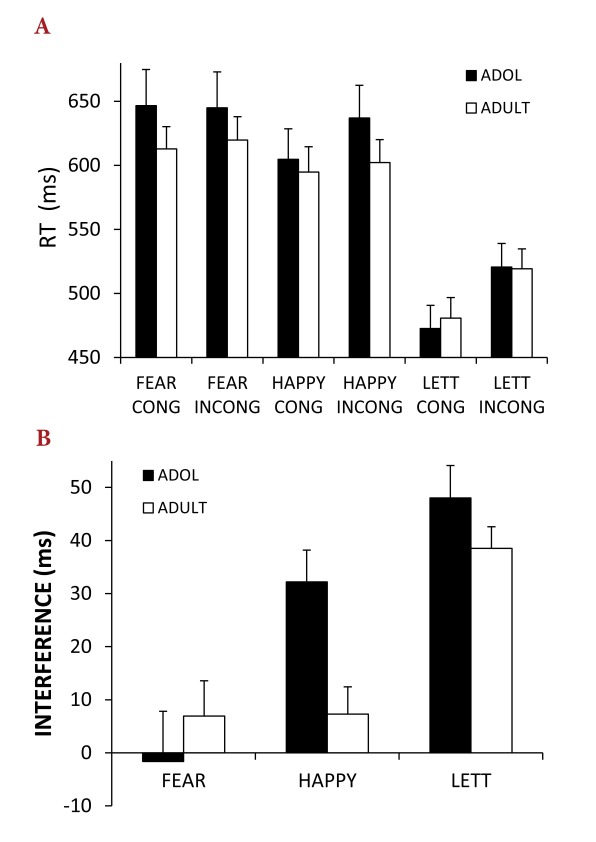
Panel A. Mean RT (in milliseconds) for each stimulus category. Panel B. Mean RTRT interference effect (in milliseconds) for each stimulus category. ADOL = adolescent. CONG = congruent. INCONG = incongruent. LETT = letter. RT= reaction time.

Panel A of Figure 3 shows the mean RT and SE for each condition for adults and adolescents for the letter task and the face task, and Panel B of Figure 3 shows the mean interference effect and SE for each condition.

#### Non-affective Letter Task

RTs for incongruent trials (*M* = 520 ms, *SD* = 84) were slower than RTs for congruent trials (*M* = 476 ms, *SD* = 85), *F*(1, 46) = 144.93, *p* < .001, η_p_^2^ = 1.00. There were no main effects of gender or age (for both, *p* > .1, η_p_^2^ < .05), and no interactions between Gender and Age (for all effects, *ps* >.1, η_p_^2^ < .04).

We also used a more conventional measure to assess age-related differences in cognitive control for the flanker task, that is, the interference effect (incongruent RT minus congruent RT). The interference effect was used as the dependent measure in an ANOVA with a between-subjects factor of Age Group (adolescent, adult), as reported above, there was no main effect of age, *F*(1, 48) = 1.67, *p* = .02, η_p_^2^ = .03.Thus, if the accuracy data is taken into account, it appears that the ability to respond rapidly and accurately to a target, even in the presence of distracting information, is relatively mature by 15 years of age when the stimuli are non-emotional.

#### Affective Face Task

RTs for incongruent trials (*M* = 626 ms, *SD* = 113) were slower than RTs for congruent trials (*M* = 614 ms, *SD* = 113), *F*(1, 46) = 12.1,*p* = .001, η_p_^2^ = .21. RTs for fearful face targets (*M* = 631 ms, *SD* = 117) were slower than for happy face targets (*M* = 609 ms, *SD* = 109), *F*(2, 92) = 13.6, *p* = .001, η_p_^2^ = .23. These main effects were qualified by a Target by Congruency interaction, *F*(2, 92) = 5.34, *p* = .03, η_p_^2^ = .10.The interference effect (i.e., increased RT for incongruent compared to congruent trials) was present for happy faces, *t*(49) = 4.6, *p* < .001, but not for fearful targets, *t* < 1. There was no main effect of age group (*F* < 1) nor any main effect or interactions with Gender (*F* < 1). However, there was a Target by Congruency by Age Group interaction, *F*(2, 92) = 5.11, *p* = .03, η_p_^2^ = .10, and a marginally significant Congruency by Age Group by Gender interaction, *F*(1, 46) = 3.43, *p* = .07, η_p_^2^ = .07. The Target by Congruency interaction was significant for adolescents, *F*(1, 23) = 8.71, *p* = .007, η_p_^2^ = .28, but not adults (F < 1).

The interference effect was used as the dependent measure in an ANOVA with within-subjects factors of Target (happy face, fearful face) and between-subjects factors of Age Group (adolescent, adult). The interference effect was greater for happy face targets (*M* = 20, *SD* = 30.3), than for fearful targets (*M* = 2.65, *SD* = 40.61), *F*(1, 48) = 5.61, *p* = .02, η_p_^2^ = .11; this was qualified by an Age Group by Target interaction, *F*(1, 48) = 5.36, *p* = .03, η_p_^2^ = .10. To tease apart the interaction between Age Group and Target, post hoc t-tests were performed for each target type. Adults and adolescents did not differ in their interference effect scores for fearful face targets, *t*(2, 48) = 0.74, *p* = .48. However, adolescents had higher interference effect scores for happy face targets than adults, *t*(2, 48) = 3.15, *p* = .003 (Bonferroni adjusted = .0125). Using the three age groups described above, we found that the interference effect for happy face targets showed a trend towards a nonlinear relationship with age, linear term, *F*(1, 49) = 6.95, *p* = .01, quadratic term, *F*(1, 49) = 2.90, *p* = .09. There was no significant difference in the interference effect between younger adolescents and older adolescents (*M*_diff_ = 4.07, *p* = .78) but interference effects were significantly greater for both groups of adolescents than adults: younger adolescents (*M*_diff_ = 22.3, *p* = .048), older adolescents (*M*_diff_ = 26.4, *p* = .005). These data suggest that the ability to successfully ignore irrelevant fearful faces and respond rapidly to a happy face, develops nonlinearly and is not yet adult-like by 15-17 years of age.

## Discussion

### Letter flanker task

In general, these results support our hypothesis that there would be minimal age-related differences between adults and adolescents in performance on a standard letter flanker task. Firstly, the RT interference effect on the letter task was similar for adolescents and adults, which indicates that the ability to react quickly to a target despite the presence of non-emotional distracting flanker stimuli matures relatively early. This finding is consistent with the behavioral results of event-related potentials (ERP) studies that used either letter (Santesso & Segalowitz, 2008) or arrow (Davies et al., 2004; Ladouceur et al., 2004, 2007) stimuli in flanker tasks with adolescents and/or children. Furthermore, we showed that accuracy on the letter task changed nonlinearly with age, such that only the youngest adolescents (< 15 years) were less accurate than the adults. Again, this result is consistent with other studies using non-affective flanker stimuli in normally developing children and adolescents, which have shown that error rates tend to be greater in younger adolescents and children, but are adult-like by 15-16 years of age (Davies et al., 2004; Ladouceur et al., 2004, 2007; Santesso & Segalowitz, 2008). Therefore, both our RT and accuracy data support the commonly reported finding that by about 15-17 years of age, the ability to ignore non-emotional distractions on a flanker task is relatively mature.

### Emotional flanker task

Our results also confirmed our hypothesis that adolescents would perform more poorly than adults on the emotional flanker task. We found that overall, adolescents made more errors than adults on the face flanker task. Accuracy on the face task improved linearly with age, regardless of the facial expression of the target or the congruency of the flankers, but was still not adult-like by 15-17 years. This suggests that the ability to accurately recognize and respond to both appetitive and aversive face stimuli, improves gradually through adolescence, that is, is age-progressive. However, the relationship between age and RT for the face task was somewhat different; adolescents took disproportionately longer than adults to respond to the target in the incongruent trials, but only when the target was a happy face. In other words, adolescents experienced greater attentional capture by fearful faces than did adults. Furthermore, the RT interference effect for happy targets flanked by fearful faces showed a non-linear relationship with age, and both younger and older adolescents showed more interference than adults. Taken together, these findings suggest that the ability to rapidly and accurately ignore distracting fearful faces has a relatively protracted developmental time-course and does not seem to be mature even by 15-17 years of age.

### Hot and cool cognitive performance in adolescents

Our finding that adolescents did relatively more poorly on the face flanker task than adults, but had comparable performance on a letter task, is consistent with other investigations of “hot” and “cool” cognitive performance in adolescents (Crone, 2009; Figner, Mackinlay, Wilkening, & Weber, 2009; Gardner & Steinberg, 2005; Hooper et al., 2004; Prencipe et al., 2011; Somerville, Hare, & Casey, 2011; Steinberg, 2007; Steinberg et al., 2008; Tottenham, Hare, & Casey, 2011). Under optimal (cool) conditions, by 15 years of age (or even earlier in some situations) adolescent performance is often adult-like; in contrast, adolescents typically perform worse than adults if the stimuli or the task places additional demands by implicating affective cognitive processing (i.e., hot cognition).

Other researchers have also shown that adolescents have relatively more difficulty ignoring emotional faces than do adults (Hare et al., 2008; Monk et al., 2003). Monk and colleagues (2003) reported no differences between adolescents (*M* = 13.12 years) and adults (*M* = 30.76 years) in terms of their accuracy and RTs when asked to evaluate a non-emotional aspect (nose width) of fearful face. However, adolescents showed greater activation than adults in brain areas associated with processing the emotional aspects of a face, which led Monk and colleagues to conclude that although there were no age-related difference in the behavioral responses, teens experienced greater involuntary attentional capture by fearful facial expressions than adults (Monk et al., 2003). Hare et al. (2008) also showed that both older adolescents (*M* = 16.0 years) and children (*M* = 9.1 years) were slower to make a go-response to fearful faces in a go/no-go task compared to adults (*M* = 23.9 years). The authors posited that this was because children andadolescents found it more difficult to over-ride the natural tendency to avoid rather than approach a fearful face than did adults. Our results are consistent with these findings: In our study, both younger and older adolescents showed more RT interference by fearful faces than adults.

Somerville, Hare, and Casey (2011) demonstrated that greater attentional capture by emotional faces in adolescents is not just restricted to faces with fearful expressions. They showed that relative to calm faces, older adolescents (*M* = 15.9 years) made more commission errors on no-go trials for happy faces than adults (*M* = 23.7 years) or children (*M* = 9.5 years).

Our results, in conjunction with those of these three functional magnetic resonance imaging (fMRI) studies, support the notion that even older adolescents are more susceptible to attentional capture by emotional faces than adults, but the relative amount of capture depends on the facial expression of the target as well as that of any competing stimuli. The ability to process both positive and negative facial emotions is of considerable importance in social settings (Williams, McGlone, Abbott, & Mattingley, 2005). Unlike Somerville, Hare, and Casey (2011), we did not show that adolescents were more distracted by happy faces than adults. However, it is important to note that Somerville and colleagues compared responses to happy and calm faces whereas we used happy and fearful faces. This suggests that regardless of valence, emotional faces are more distracting for adults than adolescents, but that fearful faces capture adolescents’ attention more effectively than happy faces. In general, research suggests that we pay more attention to negative than positive stimuli because selective attention to potentially threatening information is important for survival (Lane, Chua, & Dolan, 1999). Indeed, several studies have demonstrated an attentional bias in adults for fearful faces compared to neutral or happy faces (Eastwood, Smilek, & Merikle, 2001; Fenske & Eastwood, 2003; Hansen & Hansen, 1988; Öhman, Lundqvist, & Esteves, 2001; Smith, Cacioppo, Larsen, & Chartrand, 2003). At first glance, our results and those of others (Hare et al., 2008; Monk et al., 2003), might seem to imply that adolescents have an advantage over adults in that they are more attentive to fearful faces. However, in all of these experiments it is more adaptive to quickly realize that the fearful faces do not signal danger and that these faces can be safely ignored or approached.

We have considered the possibility that our results could be due to the fact that face-processing capabilities are still developing during adolescence (for reviews, see Batty & Taylor, 2006; Blakemore, 2008; Herba & Phillips, 2004; Scherf et al., 2011; Somerville, Fani, & McClure-Tone, 2011). We found that error rates in the face flanker task were higher overall for adolescents compared to adults, which suggests that it was relatively more difficult for adolescents to identify the facial expressions. Recognition of facial expressions has been shown to mature more quickly for happy compared to negative facial expressions, such as anger or fear (Batty & Taylor, 2006; Blakemore, 2008; Herba & Phillips, 2004; Scherf et al., 2011; Somerville, Fani, & McClure-Tone, 2011). Therefore, it is also possible that adolescents had more difficulty than adults in recognizing fearful expressions than happy ones. However, our data did not conform to these explanations: Both adolescents and adults made more errors for fearful than for happy targets, and there was no interaction between Age Group and Target Type. Furthermore, RTs and error rates were higher for adolescents when the target was a happy face flanked by fearful faces compared to fearful flanked by happy faces, which suggests that adolescents did have the ability to discriminate between the two facial expressions.

Superior performance in the recognition of facial emotions has sometimes been reported in females relative to males, even before adolescence (Scherf et al., 2011; Somerville, Fani, & McClure-Tone, 2011). However, we saw no evidence of a gender effect in our study. This may be because such gender effects are relatively subtle and may require much larger sample sizes (or a meta-analysis) to be detectable (McClure, 2000). Also, the relatively simple nature of the task (deciding between two very distinct facial expressions) may have reduced the amount of gender-related variance in our data.

As yet, we have not collected supporting electrophysiological or neuroimaging evidence to explain how behavior on our flanker tasks correlates with immaturities in underlying neural circuitry. However, other neuroimaging studies have shed light on why self-regulation is more challenging for adolescents, especially when a situation is emotionally charged or when emotional stimuli are used.

We and others have shown that by mid-adolescence, performance on self-regulation tasks under optimal “cool cognition” circumstances may be comparable to that seen in adults (Luna et al., 2010; Santesso & Segalowitz, 2008). However, neuroimaging studies reveal that this apparent behavioral maturity is not without a cost. Adolescents who perform well on these types of task either show higher levels of PFC activation than adults suggesting that they had to expend more effort (Luna, 2009) or activate a wider area of cortical tissue than seen in adults (Durston et al., 2006) in order to produce a comparable result. In either case, it would appear that even under low-arousal circumstances, the self-regulatory system is somewhat taxed in adolescents. Therefore, the addition of an affective component to a task, might well result in less effective cognitive control in adolescents.

Along these lines, it has been suggested that it is the imbalance between the maturation of the limbic system compared to the PFC that increases adolescents’ vulnerability to poor cognitive control in situations or tasks that have an affective context (Casey et al., 2008; Galvan et al., 2005; Hare et al., 2008). The limbic system, which drives emotionally motivated behaviors, shows hyperactivation to both positive and negative emotional stimuli in adolescents in comparison to children and adults (Ernst, Pine, & Hardin, 2006; Guyer et al., 2008; Hare et al., 2008; Monk et al., 2003). Therefore, it appears that adolescence is a time of heightened arousal to emotional stimuli. In contrast, the PFC and other associated frontal cortical areas such as the anterior cingulate, which are important for the regulation of emotionally driven activity, are relatively underdeveloped in adolescents compared to adults (Hare et al., 2008). Therefore, under optimal “cool cognition” circumstances, adolescents can probably recruit additional brain areas or produce greater activation in the PFC to appear adult-like, but adding an affective component to a task or a situation is more likely to overload the system, and lead to impaired performance.

### Directions for future research

Our study was a preliminary investigation to ascertain whether a face flanker task was an effective “hot” cognitive task for demonstrating developmental differences in cognitive control between adults and adolescents, in preparation for a more extensive ERP study. As such, there are several parameters that we did not measure that might be useful to include in future studies. Firstly, we did not measure pubertal status, and so were unable to investigate whether, as suggested by others (e.g., Forbes, Phillips, Silk, Ryan, & Dahl, 2011), pubertal status is a better predictor of cognitive performance with affective stimuli than age. Secondly, we did not plan to examine age-related changes in performance across childhood into adolescence and so did not include a group of younger children. Therefore, our data on the developmental trajectory of attentional capture by fearful faces should be considered preliminary. Furthermore, we cannot establish whether the interference effect peaks in adolescence, or if it also declines between childhood and adolescence. Larger studies that included younger children have shown adolescent-specific increases both in behavioral responsivity to various appetitive stimuli (e.g., Burnett et al., 2010; Cauffman et al., 2010; Somerville, Hare, & Casey, 2011), and in neural activity (but not behavior) to fearful faces (e.g., Hare et al., 2008) and large losses (for a review, see Ernst & Fudge, 2009).

We posit that if teenagers find it more difficult to suppress irrelevant emotional faces than adults, this may make them more vulnerable in situations where other types of emotional distractions need to be ignored in order to make effective and safe decisions. Because the main goal of the study was to investigate group-level differences between adults and adolescents, we did not assess how individual differences in risk taking behaviors correlated with task performance.

In conclusion, this study shows that the ability to self-regulate in teenagers, as assessed by the ability to suppress goal-irrelevant information in a flanker task, is dependent on the affective nature of the stimuli. Performance on a non-emotional (letter) flanker task was comparable for adults and teenagers by 15-17 years. However, fearful emotional faces were more difficult for adolescents to ignore, even when they were irrelevant to the central task, suggesting that these expressions captured the attention of adolescents more strongly. Further research is necessary to determine whether this heightened sensitivity to fearful faces may reflect a more generalizable distractibility to social emotional stimuli that leads to increased risk taking in adolescents.

## Author Note

Tina Zottoli is now at St. Joseph’s College of New York.

This research was supported in part by a PSC-CUNY grant and a John Jay College Research Assistance grant awarded to Jillian Grose-Fifer.

We thank OzlemYuksel-Sokmen for her help with the pilot studies that led to this study, and Danielle Mascarelli and Michelle Winokur for their assistance with preliminary data analyses.

## References

[R1] Adleman N. E., Menon V., Blasey C. M., White C. D., Warsofsky I. S., Glover G. H. (2002). A developmental fMRI study of the Stroop color-word
task.. NeuroImage.

[R2] Batty M., Taylor M. J. (2006). The development of emotional face processing during
childhood.. Developmental Science.

[R3] Blakemore S.-J. (2008). The social brain in adolescence.. Nature Reviews Neuroscience.

[R4] Blakemore S.-J., Choudhury S. (2006). Development of the adolescent brain: Implications for executive
function and social cognition.. Journal of Child Psychology and Psychiatry.

[R5] Bunge S. A., Hazeltine E., Scanlon M. D., Rosen A. C., Gabrieli J. D. E. (2002). Dissociable contributions of prefrontal and parietal cortices to
response selection.. NeuroImage.

[R6] Burnett S., Bault N., Coricelli G., Blakemore S.-J. (2010). Adolescents’ heightened risk-seeking in a probabilistic
gambling task.. Cognitive Development.

[R7] Casey B. J., Jones R. M., Hare T. A. (2008). The adolescent brain.. Annals of the New York Academy of Sciences.

[R8] Casey B. J., Jones R. M., Levita L., Libby V., Pattwell S. S., Ruberry E. J. (2010). The storm and stress of adolescence: Insights from human imaging
and mouse genetics.. Developmental Psychobiology.

[R9] Casey B. J., Trainor R. J., Orendi J. L., Schubert A. B., Nystrom L. E., Giedd J. N. (1997). A developmental functional MRI study of prefrontal activation
during performance of a go-no-go task.. Journal of Cognitive Neuroscience.

[R10] Cauffman E., Shulman E. P., Steinberg L., Claus E., Banich M. T., Graham S. (2010). Age differences in affective decision making as indexed by
performance on the Iowa gambling task.. Developmental Psychology.

[R11] Chein J., Albert D., O’Brien L., Uckert K., Steinberg L. (2011). Peers increase adolescent risk taking by enhancing activity in
the brain’s reward circuitry.. Developmental Science.

[R12] Crone E. A. (2009). Executive functions in adolescence: Inferences from brain and
behavior.. Developmental Science.

[R13] Crone E. A., Bullens L., van der Plas E. A. A., Kijkuit E. J., Zelazo P. D. (2008). Developmental changes and individual differences in risk and
perspective taking in adolescence.. Development and Psychopathology.

[R14] Davies P. L., Segalowitz S. J., Gavin W. J. (2004). Development of response-monitoring ERPs in 7- to
25-year-olds.. Developmental Neuropsychology.

[R15] Durston S., Davidson M. C., Tottenham N., Galvan A., Spicer J., Fossella J. A. (2006). A shift from diffuse to focal cortical activity with
development.. Developmental Science.

[R16] Eastwood J., Smilek D., Merikle P. (2001). Differential attentional guidance by unattended faces expressing
positive and negative emotion.. Attention, Perception, and Psychophysics.

[R17] Eriksen B. A., Eriksen C. W. (1974). Effects of noise letters upon the identification of a target
letter in a nonsearch task.. Perception & Psychophysics.

[R18] Ernst M., Fudge J. L. (2009). A developmental neurobiological model of motivated behavior:
Anatomy, connectivity, and ontogeny of the triadic nodes.. Neuroscience and Biobehavioral Reviews.

[R19] Ernst M., Pine D. S., Hardin M. (2006). Triadic model of the neurobiology of motivated behavior in
adolescence.. Psychological Medicine.

[R20] Fenske M. J., Eastwood J. D. (2003). Modulation of focused attention by faces expressing emotion:
Evidence from flanker tasks.. Emotion.

[R21] Figner B., Mackinlay R. J., Wilkening F., Weber E. U. (2009). Affective and deliberative processes in risky choice: Age
differences in risk taking in the Columbia card task.. Journal of Experimental Psychology: Learning, Memory, and
Cognition.

[R22] Forbes E. E., Phillips M. L., Silk J. S., Ryan N. D., Dahl R. A. (2011). Neural systems of threat processing in adolescents: Role of
pubertal maturation and relation to measures of negative
affect.. Developmental Neuropsychology.

[R23] Galvan A., Hare T. A., Davidson M., Spicer J., Glover G., Casey B. J. (2005). The role of ventral frontostriatal circuitry in reward-based
learning in humans.. Journal of Neuroscience.

[R24] Gardner M., Steinberg L. (2005). Peer influence on risk taking, risk preference, and risky
decision making in adolescence and adulthood: An experimental
study.. Developmental Psychology.

[R25] Geier C., Luna B. (2009). The maturation of incentive processing and cognitive
control.. Pharmacology Biochemistry and Behavior.

[R26] Giedd J. N. (2004). Structural magnetic resonance imaging of the adolescent
brain.. Annals of the New York Academy of Science.

[R27] Guyer A. E., Monk C. S., McClure-Tone E. B., Nelson E. E., Roberson-Nay R., Adler A. D. (2008). A developmental examination of amygdala response to facial
expressions.. Journal of Cognitive Neuroscience.

[R28] Hansen C. H., Hansen R. D. (1988). Finding the face in the crowd: An anger superiority
effect.. Journal of Personality and Social Psychology.

[R29] Hare T. A., Tottenham N., Galvan A., Voss H. U., Glover G. H., Casey B. J. (2008). Biological substrates of emotional reactivity and regulation in
adolescence during an emotional go-nogo task.. Biological Psychiatry.

[R30] Herba C., Phillips M. (2004). Annotation: Development of facial expression recognition from
childhood to adolescence: Behavioural and neurological
perspectives.. Journal of Child Psychology and Psychiatry.

[R31] Hooper C. J., Luciana M., Conklin H. M., Yarger R. S. (2004). Adolescents’ performance on the Iowa gambling task:
Implications for the development of decision making and ventromedial
prefrontal cortex.. Developmental Psychology.

[R32] Joormann J., Talbot L., Gotlib I. H. (2007). Biased processing of emotional information in girls at risk for
depression.. Journal of Abnormal Psychology.

[R33] Ladouceur C. D., Dahl R. E., Carter C. S. (2004). ERP correlates of action monitoring in
adolescence.. Annals of the New York Academy of Sciences.

[R34] Ladouceur C. D., Dahl R. E., Carter C. S. (2007). Development of action monitoring through adolescence into
adulthood: ERP and source localization.. Developmental Science.

[R35] Lamm C., Zelazo P. D., Lewis M. D. (2006). Neural correlates of cognitive control in childhood and
adolescence: Disentangling the contributions of age and executive
function.. Neuropsychologia.

[R36] Lane R. D., Chua P. M. L., Dolan R. J. (1999). Common effects of emotional valence, arousal, and attention on
neural activation during visual processing of pictures.. Neuropsychologia.

[R37] Lavie N., De Fockert J. (2005). The role of working memory in attentional
capture.. Psychonomic Bulletin & Review.

[R38] Lazarus R. S., Smith C. A. (1988). Knowledge and appraisal in the cognition-emotion
relationship.. Cognition & Emotion.

[R39] Luna B. (2009). Developmental changes in cognitive control through
adolescence.. Advances in Child Development and Behavior.

[R40] Luna B., Padmanabhan A., O’Hearn K. (2010). What has fMRI told us about the development of cognitive control
through adolescence?. Brain and Cognition.

[R41] Luna B., Thulborn K. R., Munoz D. P., Merriam E. P., Garver K. E., Minshew N. J. (2001). Maturation of widely distributed brain function subserves
cognitive development.. NeuroImage.

[R42] McClure E. B. (2000). A meta-analytic review of sex differences in facial expression
processing and their development in infants, children, and
adolescents.. Psychological Bulletin.

[R43] Monk C. S., McClure E. B., Nelson E. E., Zarahn E., Bilder R. M., Leibenluft E. (2003). Adolescent immaturity in attention-related brain engagement to
emotional facial expressions.. NeuroImage.

[R44] Moser J. S., Huppert J. D., Duval E., Simons R. F. (2008). Face processing biases in social anxiety: An electrophysiological
study.. Biological Psychology.

[R45] Munro G. E. S., Dywan J., Harris G. T., McKee S., Unsal A., Segalowitz S. J. (2007). ERN varies with degree of psychopathy in an emotion
discrimination task.. Biological Psychology.

[R46] Nigg J. T. (2000). On inhibition/disinhibition in developmental psychopathology:
Views from cognitive and personality psychology and a working inhibition
taxonomy.. Psychological Bulletin.

[R47] Ochsner K. N., Hughes B., Robertson E. R., Cooper J. C., Gabrieli J. D. E. (2009). Neural systems supporting the control of affective and cognitive
conflicts.. Journal of Cognitive Neuroscience.

[R48] Öhman A., Lundqvist D., Esteves F. (2001). The face in the crowd revisited: A threat advantage with
schematic stimuli.. Journal of Personality and Social Psychology.

[R49] Overman W. H., Frassrand K., Ansel S., Trawalter S., Bies B., Redmond A. (2004). Performance on the IOWA card task by adolescents and
adults.. Neuropsychologia.

[R50] Padmanabhan A., Geier C. F., Ordaz S. J., Teslovich T., Luna B. (2011). Developmental changes in brain function underlying the influence
of reward processing on inhibitory control.. Developmental Cognitive Neuroscience.

[R51] Prencipe A., Kesek A., Cohen J., Lamm C., Lewis M. D., Zelazo P. D. (2011). Development of hot and cool executive function during the
transition to adolescence.. Journal of Experimental Child Psychology.

[R52] Rivers S. E., Reyna V. F., Mills B. A. (2008). Risk taking under the influence: A fuzzy-trace theory of emotion
in adolescence.. Developmental Review.

[R53] Rubia K., Smith A. B., Taylor E., Brammer M. (2007). Linear age-correlated functional development of right inferior
fronto-striato-cerebellar networks during response inhibition and anterior
cingulate during error-related processes.. Human Brain Mapping.

[R54] Santesso D. L., Segalowitz S. J. (2008). Developmental differences in error-related ERPs in middle- to
late-adolescent males.. Developmental Psychology.

[R55] Scherf K. S., Behrmann M., Dahl R. E. (2011). Facing changes and changing faces in adolescence: A new model for
investigating adolescent-specific interactions between pubertal, brain, and
behavioral development.. Developmental Cognitive Neuroscience.

[R56] Smith N. K., Cacioppo J. T., Larsen J. T., Chartrand T. L. (2003). May I have your attention, please: Electrocortical responses to
positive and negative stimuli.. Neuropsychologia.

[R57] Somerville L. H., Fani N., McClure-Tone E. B. (2011). Behavioral and neural representation of emotional facial
expressions across the lifespan.. Developmental Neuropsychology.

[R58] Somerville L. H., Hare T., Casey B. J. (2011). Frontostriatal maturation predicts cognitive control failure to
appetitive cues in adolescents.. Journal of Cognitive Neuroscience.

[R59] Somerville L. H., Jones R. M., Casey B. J. (2010). A time of change: Behavioral and neural correlates of adolescent
sensitivity to appetitive and aversive environmental cues.. Brain and Cognition.

[R60] Steinberg L. (2007). Risk taking in adolescence: New perspectives from brain and
behavioral science.. Current Directions in Psychological Science.

[R61] Steinberg L. (2008). A social neuroscience perspective on adolescent
risk-taking.. Developmental Review.

[R62] Steinberg L. (2010). A dual systems model of adolescent risk-taking.. Developmental Psychobiology.

[R63] Steinberg L., Albert D., Cauffman E., Banich M., Graham S., Woolard J. (2008). Age differences in sensation seeking and impulsivity as indexed
by behavior and self-report: Evidence for a dual systems
model.. Developmental Psychology.

[R64] Thomas K. M., Drevets W. C., Dahl R. E., Ryan N. D., Birmaher B., Eccard C. H. (2001). Amygdala response to fearful faces in anxious and depressed
children.. Archives of General Psychiatry.

[R65] Thomas L. A., De Bellis M. D., Graham R., LaBar K. S. (2007). Development of emotional facial recognition in late childhood and
adolescence.. Developmental Science.

[R66] Tottenham N., Hare T. A., Casey B. J. (2011). Behavioral assessment of emotion discrimination, emotion
regulation, and cognitive control in childhood, adolescence, and
adulthood.. Frontiers in Developmental Psychology.

[R67] Tottenham N., Tanaka J. W., Leon A., McCarry T., Nurse M., Hare T. A. (2009). The NimStim set of facial expressions: Judgments from untrained
research participants.. Psychiatry Research.

[R68] van Boxtel G. J. M., van der Molen M. W., Jennings J. R., Brunia C. H. M. (2001). A psychophysiological analysis of inhibitory motor control in the
stop-signal paradigm.. Biological Psychology.

[R69] van Duijvenvoorde A. C. K., Jansen B. R. J., Visser I., Huizenga H. M. (2010). Affective and cognitive decision-making in
adolescents.. Developmental Neuropsychology.

[R70] Van Leijenhorst L., Moor B. G., Op de Macks Z. A., Rombouts S. A. R. B., Westenberg P. M., Crone E. A. (2010). Adolescent risky decision-making: Neurocognitive development of
reward and control regions.. NeuroImage.

[R71] Velanova K., Wheeler M. E., Luna B. (2008). Maturational changes in anterior cingulate and frontoparietal
recruitment support the development of error processing and inhibitory
control.. Cerebral Cortex.

[R72] Williams M. A., McGlone F., Abbott D. F., Mattingley J. B. (2005). Differential amygdala responses to happy and fearful facial
expressions depend on selective attention.. NeuroImage.

